# Sex-dependent effects of ambient PM_2.5_ pollution on insulin sensitivity and hepatic lipid metabolism in mice

**DOI:** 10.1186/s12989-020-00343-5

**Published:** 2020-04-22

**Authors:** Ran Li, Qing Sun, Sin Man Lam, Rucheng Chen, Junyao Zhu, Weijia Gu, Lu Zhang, He Tian, Kezhong Zhang, Lung-Chi Chen, Qinghua Sun, Guanghou Shui, Cuiqing Liu

**Affiliations:** 1 School of Basic Medical Sciences and Public Health, Zhejiang Chinese Medical University, 548 Binwen Rd, Building 15#, Room 215, Hangzhou, 310053 China; 2 Joint China-US Research Center for Environment and Pulmonary Diseases, Zhejiang Chinese Medical University, Hangzhou, China; 3 State Key Laboratory of Molecular Developmental Biology, Institute of Genetics and Developmental Biology, Chinese Academy of Sciences, 1 West Beichen Rd, Building 2, Room 306, Beijing, 100101 China; 4 Center for Molecular Medicine and Genetics, Wayne State University School of Medicine, Detroit, MI USA; 5 Department of Environmental Medicine, New York University of School of Medicine, New York, USA; 6 College of Public Health, The Ohio State University, Columbus, OH USA

**Keywords:** Sex difference, Air pollution, Insulin resistance, Lipids accumulation, Lipidomics

## Abstract

**Background & aims:**

Emerging evidence supports ambient fine particulate matter (PM_2.5_) exposure is associated with insulin resistance (IR) and hepatic lipid accumulation. In this study, we aimed to evaluate the sex-dependent vulnerability in response to PM_2.5_ exposure and investigate the underlying mechanism by which PM_2.5_ modulates hepatic lipid metabolism.

**Methods:**

Both male and female C57BL/6 mice were randomly assigned to ambient PM_2.5_ or filtered air for 24 weeks via a whole body exposure system. High-coverage quantitative lipidomics approaches and liquid chromatography-mass spectrometry techniques were performed to measure hepatic metabolites and hormones in plasma. Metabolic studies, histological analyses, as well as gene expression levels and molecular signal transduction analysis were applied to examine the effects and mechanisms by which PM_2.5_ exposure-induced metabolic disorder.

**Results:**

Female mice were more susceptible than their male counterparts to ambient PM_2.5_ exposure-induced IR and hepatic lipid accumulation. The hepatic lipid profile was changed in response to ambient PM_2.5_ exposure. Levels of hepatic triacylglycerols (TAGs), free fatty acids (FFAs) and cholesterol were only increased in female mice from PM group compared to control group. Plasmalogens were dysregulated in the liver from PM_2.5_-exposed mice as well. In addition, exposure to PM_2.5_ led to enhanced hepatic ApoB and microsomal triglyceride transport protein expression in female mice. Finally, PM_2.5_ exposure inhibited hypothalamus-pituitary-adrenal (HPA) axis and decreased glucocorticoids levels, which may contribute to the vulnerability in PM_2.5_-induced metabolic dysfunction.

**Conclusions:**

Ambient PM_2.5_ exposure inhibited HPA axis and demonstrated sex-associated differences in its effects on IR and disorder of hepatic lipid metabolism. These findings provide new mechanistic evidence of hormone regulation in air pollution-mediated metabolic abnormalities of lipids and more personalized care should be considered in terms of sex-specific risk factors.

## Background

Recent evidence indicates an association between air pollution and diabetes risk, including whole body insulin resistance (IR), lipid accumulation and glucose metabolism dysfunction [1–4]. As a critical target organ of metabolism, liver pathogenesis in response to PM_2.5_ (particulate matter ≤2.5 μm) might shed light on the mechanism of such metabolic disorders. Our previous observations demonstrated that PM_2.5_ exposure led to hepatic IR that was accompanied with fatty liver [5], non-alcoholic steatohepatitis, and impaired hepatic glucose metabolism [6–8]. However, the molecular mechanism and specific lipid metabolic signaling pathway responsible for the PM_2.5_-mediated metabolic disorder in the liver are poorly understood.

There is increasing evidence that sex is an important factor in epidemiology and pathophysiology of diabetes. Type-2 diabetes is more frequently diagnosed in younger age and lower body mass index particularly in men, whereas the most prominent risk factor of obesity is more common in women [9]. However, a number of pertinent studies reported more pronounced effects of air pollution exposure on diabetes prevalence/incidence in women than in men [10–13]. A meta-analysis of 13 studies in Europe and North America demonstrated higher sensitivity to PM_2.5_ exposure in females as well [14]. However, to date, no animal study has been pursued to define the mechanism underlying sex difference in vulnerability to environmental risk factors in the development of metabolic syndrome.

Lipids are essential metabolites that are required for the basic cellular functions and can provide a global readout metabolic status of cells. Lipidomics emerged as an approach which quantifies the alteration of individual lipid classes and species, offers promising lipid biomarkers and precious information which enables us to study cellular metabolic differences in response to stimulation. Lipid metabolites have been shown to be closely associated with metabolic diseases, cardiovascular, neurodegenerative and respiratory diseases, and cancer. Thus, elucidating lipidome response to PM_2.5_ has important implications in the understanding of the molecular basis of air pollution and the development of these disorders. To our knowledge, however, very little is known with regard to lipid metabolism in the liver in response to environmental PM_2.5_ exposure.

In this study, we systematically examined the sex difference in air pollution-mediated IR and lipid in the liver and circulating steroid hormones. Through lipidomics analyses, we provide a comprehensive insight into the effects and mechanistic basis of ambient real-world PM_2.5_ exposure on IR and hepatic lipid metabolism and characterize the sex-dependent susceptibility to PM_2.5_ exposure. In addition, PM_2.5_ exposure inhibited hypothalamus-pituitary-adrenal (HPA) axis and glucocorticoids levels, which may contribute to the vulnerability in PM_2.5_-induced metabolic dysfunction. These findings provide new mechanistic evidence of hormone regulation and lipid metabolites in air pollution-mediated metabolic abnormalities and more personalized care should be considered in terms of sex-specific risk factors.

## Results

### PM_2.5_ concentration and compositional assessment

**Fig. 1:**
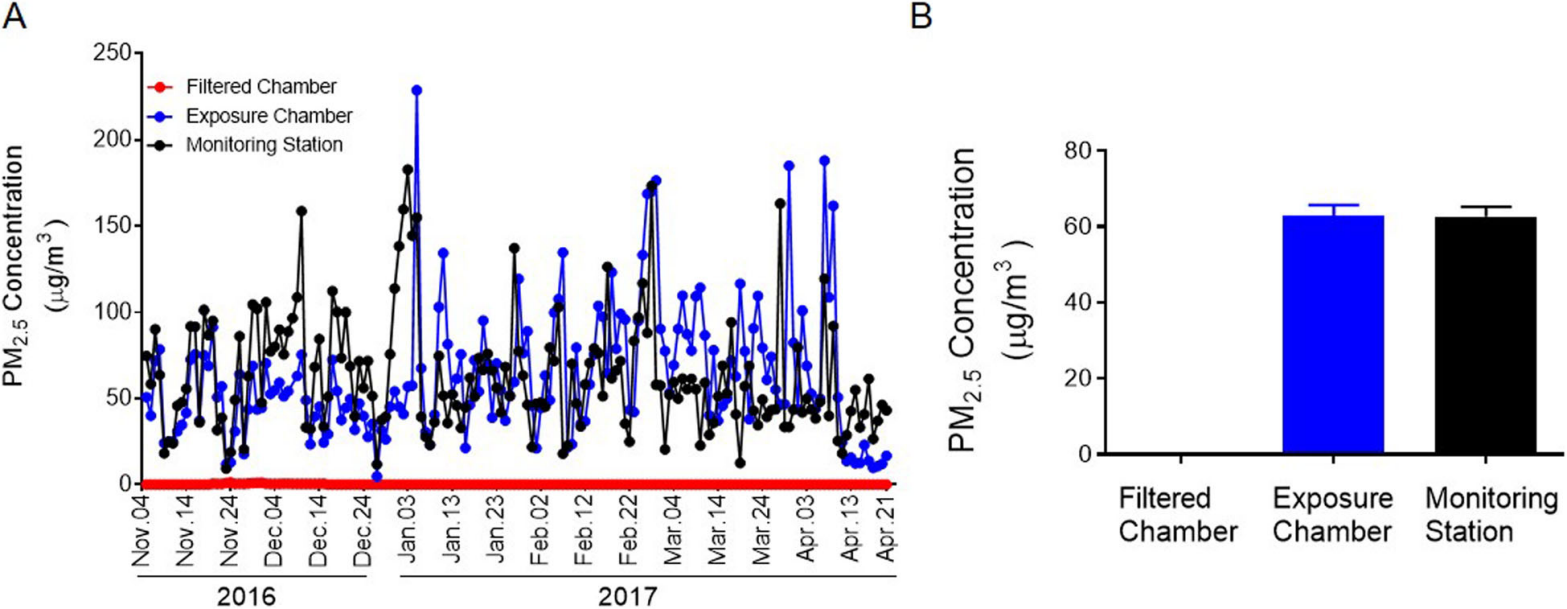
PM_2.5_ concentrations at the study site. **A**, PM_2.5_ concentrations in the filter chamber, exposed chamber and ambient air from the monitoring station during the exposure period. **B**, Bar graph of mean daily PM_2.5_ concentrations in the filter chamber, exposed chamber and ambient air from the monitoring station during the exposure period

The exposure period covered seasons of winter (2016) and spring (2017). The PM_2.5_ levels in the exposure chamber and in the ambient air were correlated while the maximum values were 228.70 μg/m^3^ and 182.70 μg/m^3^, and minimum values 4.70 μg/m^3^ and 9.27 μg/m^3^ in the exposure chamber and in the ambient air, respectively (Fig. 1a). The regression coefficient between exposure chamber and ambient air concentrations was 0.365 (*P* < 0.001). Mean daily PM_2.5_ concentrations in the ambient air at the study site was 62.63 μg/m^3^ (SEM, 2.60 μg/m^3^), while mean daily concentrations of PM_2.5_ in the filtered chamber and exposure chamber were 0.09 μg/m^3^ (SEM, 0.02 μg/m^3^) and 62.74 μg/m^3^ (SEM, 2.96 μg/m^3^), respectively (Fig. 1b). The mean daily concentrations in the exposure chamber were very closed to the ones in the ambient air at the study site during the same time period (both were ranged between 60 and 70 μg/m^3^) and the maximum concentrations of 228.70 μg/m^3^ in the exposure chamber, although 46 μg/m^3^ higher than the ambient one (182.70 μg/m^3^), is frequently seen during peak hours in major cities in China. Elemental composition was demonstrated in Supplemental Material, Table S1.

### Ambient PM_2.5_ exposure decreased insulin sensitivity, being more prominent in female than in male

**Fig. 2:**
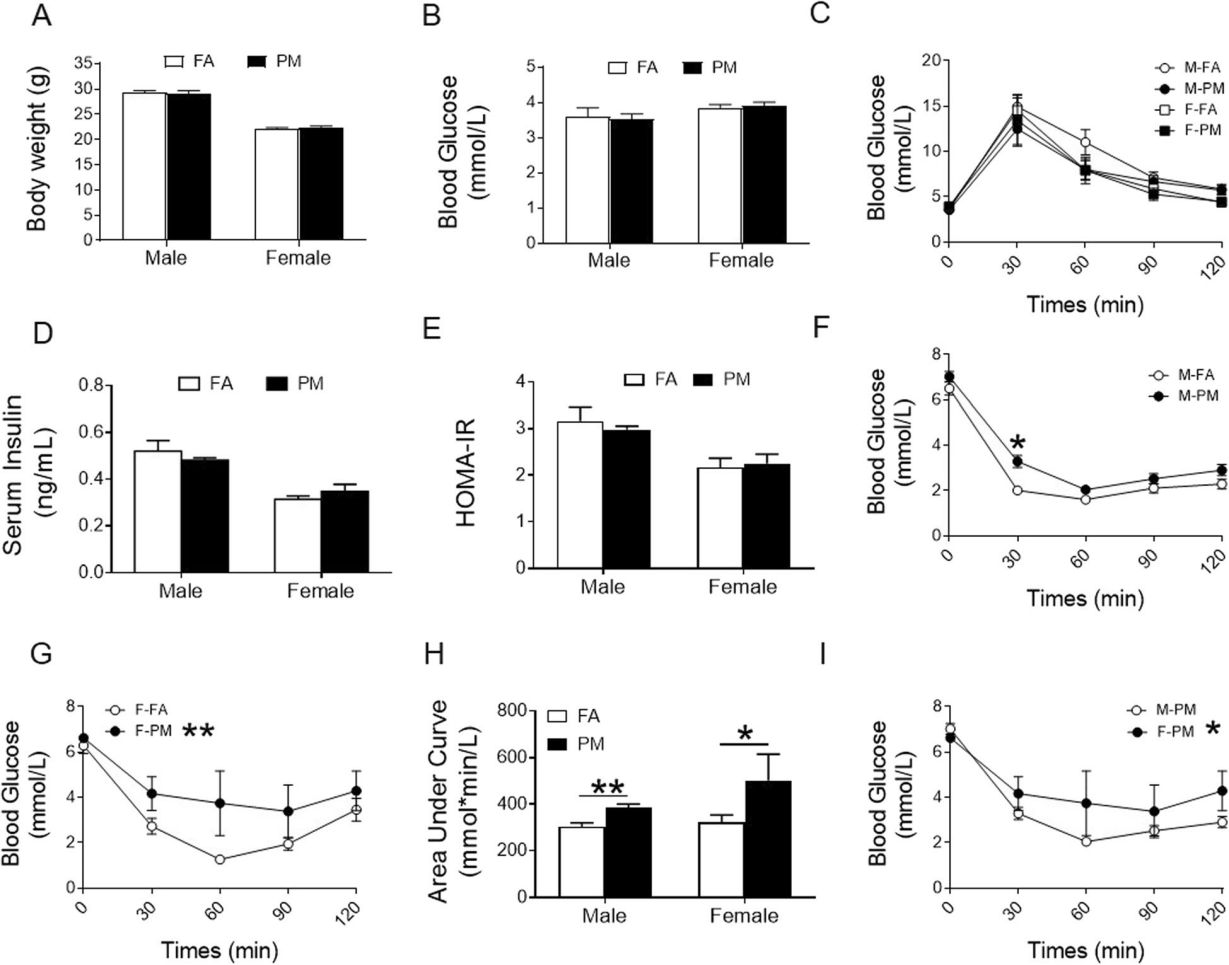
Effects of PM_2.5_ exposure on body weight and glucose homeostasis in C57BL/6 mice. **A**, Body weight of mice at the end of PM_2.5_ exposure. **B**, Fasting blood glucose at the end of PM_2.5_ exposure. **C**, GTT in fasted mice at the end of PM_2.5_ exposure. **D** and **E**, Fasting insulin levels and HOMA-IR at the end of PM_2.5_ exposure. **F**-**G**, ITT in fasted male (F) and female (G) mice at the end of PM_2.5_ exposure. **H**, ITT analysis with AUC (area under curves). **I**, ITT in fasted male and female mice exposed to PM_2.5_. *P < 0.05, **P < 0.01 for each comparison. * beside the legend in the figure panel denotes overall difference. *n* = 5-8

Prior to assignment to exposure protocols, no significant difference between groups at baseline in body weight, fasting blood glucose within male or female mice was observed (Supplemental Fig. 1, A-D). After 24 weeks of PM_2.5_ exposure, we observed no effects of PM_2.5_ exposure on body weight (Fig. 2a, Supplemental Fig. 2A), blood glucose (Fig. 2b, Supplemental Fig. 2B), glucose tolerance (Fig. 2c), serum insulin levels (Fig. 2d, Supplemental Fig. 2C) or the homeostasis model assessment of the IR (HOMA-IR, Supplemental Fig. 2D) index (Fig. 2e) in each sex. Nevertheless, PM_2.5_-exposed mice displayed attenuation of whole-body insulin sensitivity in response to intraperitoneal insulin injection (Fig. 2h, Supplemental Fig. 2E), evidenced by higher blood glucose 30 min after insulin injection in male mice (Fig. 2f) and clear separation of insulin tolerance test (ITT) curves in FA and PM groups in female mice (Fig. 2g). Interestingly, insulin sensitivity was worse in PM_2.5_^-^exposed female mice than that in male mice (Fig. 2i). Taken together, these results suggest that PM_2.5_-induced dysregulation in whole body insulin sensitivity but not in control of post-prandial glycemic response. In addition, sex-dependence was observed for PM_2.5_-associated attenuation in insulin sensitivity, with female mice being more susceptible

### Ambient PM_2.5_ exposure induced hepatic lipid deposition in female mice

**Fig. 3:**
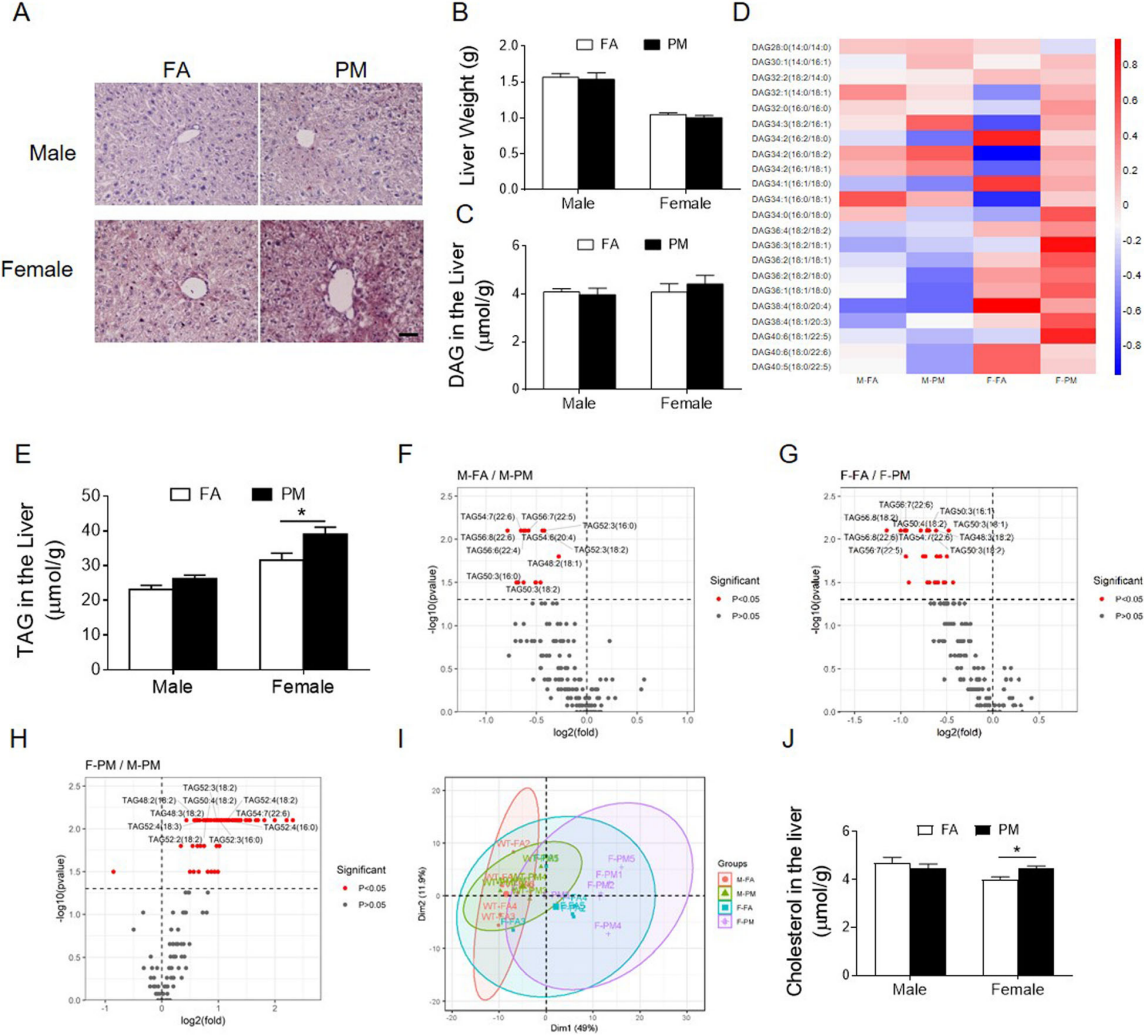
Effects of PM_2.5_ exposure on neutral lipids in the liver. **A** and **B**, Representative images of oil red O staining of liver sections (A) and liver organ weight (B). scale bar = 100 μm. **C** and **D**, Total DAG levels (C) and Hierarchical clustering heatmap analysis of DAG lipids (D) in the hepatic lipid extracts from mice. **E**, Total TAG levels in the hepatic lipid extracts from mice. **F**-**H**, Volcano plots of all TAG species detected in the liver from male mice exposed to FA and PM_2.5_ (F), from female mice exposed to FA and PM_2.5_ (G) and from PM_2.5_-exposed male and female mice (H). *P* < 0.05 is in red. **I**, PCA analysis of whole lipidome of male and female mice exposed to FA or PM_2.5_. **J** and **K**, Major species of CE with significant difference in the hepatic lipid extracts from male (J) and female (K) mice. *P < 0.05 for each comparison. n = 5 for lipidomics study and *n* = 7–8 for oil red O examination

To get a more comprehensive understanding on the effect of PM_2.5_ on organ metabolism, hepatic lipid deposition was examined with Oil Red O staining. As shown in Fig. 3, PM_2.5_^-^exposed mice displayed more intracytoplasmic lipids in female than male mice (Fig. 3a), but we observed no effects of PM_2.5_ on liver mass (Fig. 3b).

Next, high-coverage quantitative lipidomics analysis was conducted on liver samples collected from both FA and PM_2.5_ exposed mice. A total of 421 lipids species from 24 subclasses were quantified. Although we observed no significant difference in either total hepatic diacylglycerols (DAG) or individual lipid species (Fig. 3, Supplemental Fig. 3A), a significant increase in levels of triacylglycerols (TAGs) was observed in PM exposed female mice but not in male mice (Fig. 3e, Supplemental Fig. 3B). Then, individual lipid species were examined. PM_2.5_ exposure induced significant increase in 15 of the 105 TAG species in male mice (Fig. 3f, Supplemental Material, Table S2). They were three saturated fatty acids (SFAs) of palmitic acid (16:0)-containing TAG, two docosahexaenoic acid (22:6)-containing TAG, two monounsaturated fatty acids (MUFAs) (16:1, 18:1)-containing TAG, and eight polyunsaturated fatty acids (PUFAs) (18:2, 18:3, 20:4, 22:5)-containing TAG (Supplemental Material, Table S2). PM_2.5_ exposure induced significant increase in 27 of the 105 TAG species in female mice (Fig. 3g, Supplemental Material, Table S2). They were four SFA of palmitic acid (16:0)-containing TAG, five DHA (22:6)-containing TAG, four MUFA (16:1, 18:1)-containing TAG, and 14 PUFA (16:2, 18:2, 18:3, 20:4, 22:5)-containing TAG (Supplemental Material, Table S2). Interestingly, consistent with the total level of TAG which showing higher level in female mice than male mice in response to PM_2.5_ exposure, 64 of the 105 TAG species increased in female mice compared to male mice (Fig. 3h, Supplemental Material, Table S2). The sex difference in response to PM_2.5_ exposure was further emphasized by principal component analysis (PCA), which discriminated the data obtained in ovals and circles (Fig. 3i). In addition, we also found a significant increase in levels of hepatic free cholesterols in PM_2.5_ exposed female mice but not in male mice (Fig. 3j, Supplemental Fig. 3C). There was no significant difference in levels of total cholesterol ester (CE) or CE species (Supplemental Fig. 3D, G, E and H) in response to PM_2.5_ exposure in male mice, whereas 2 of 18 CE species (CE-16:1, CE-20:3) elevated in PM_2.5_ exposed female mice compared to FA exposed female mice (Supplemental Fig. 3F and I). These observations cumulatively suggest a PM_2.5_-induced lipid accumulation in the liver, in particular in female mice.

### Ambient PM_2.5_ exposure changes the profile of fatty acids in the liver

**Fig. 4:**
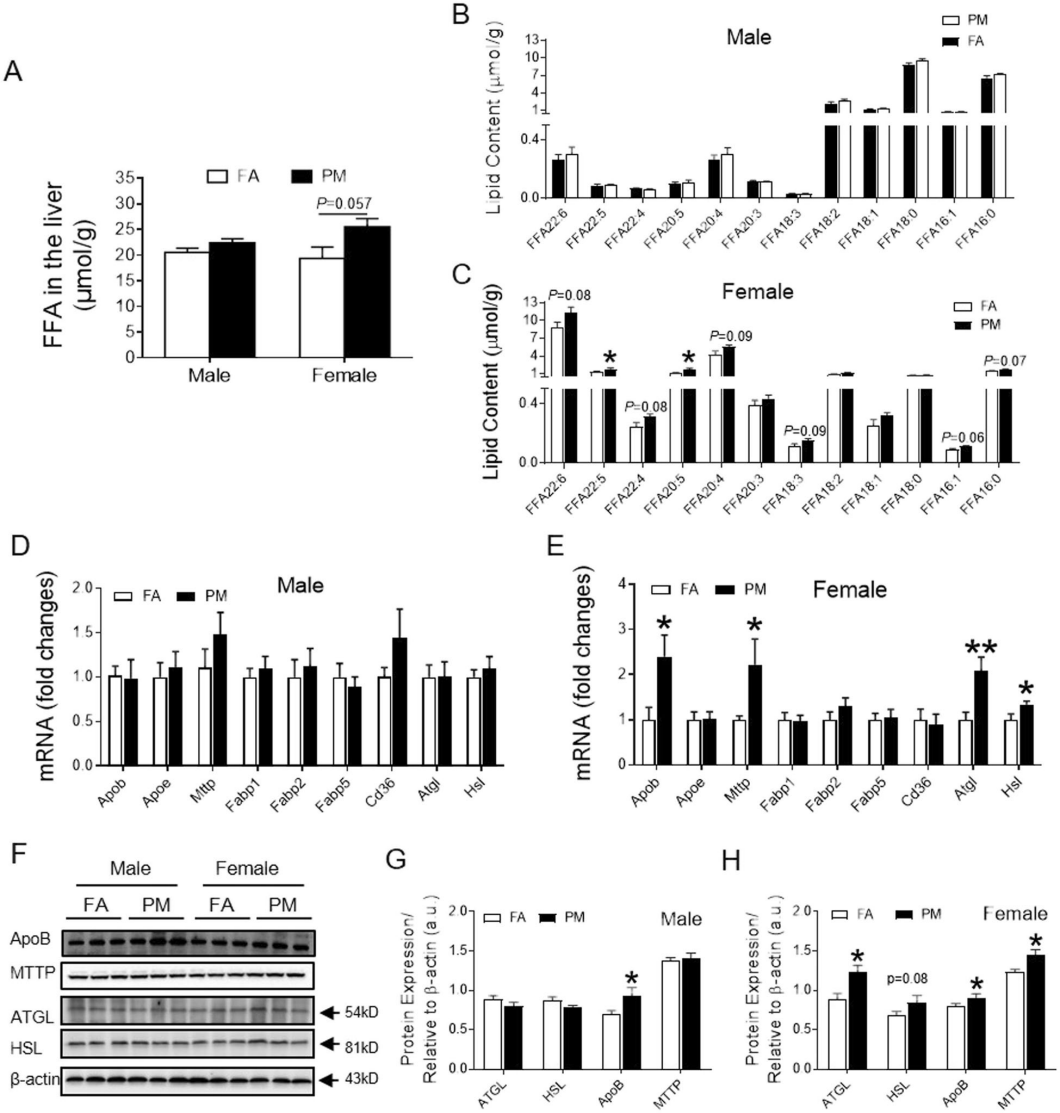
Effects of PM_2.5_ exposure on FFA profile and relevant signals in the liver. **A**, Total FFA content in the hepatic lipid extracts in mice. **B** and **C**, Major species of FFA in the hepatic lipid extracts from male (B) and female (C) mice. **D** and **E**, mRNA levels of *Apob, Apoe, Mttp,* enzymes involved in lipolysis and fatty acid uptake in the liver from male (D) and female (E) mice. **F**-**H**, Representative bands (F) and analyzed protein levels of ApoB, MTTP, ATGL and HSL in the liver tissue from male (G) and female (H) mice. *P < 0.05, **P < 0.01 for each comparison. n = 5 for lipidomic analysis, n = 7–9 for mRNA examination, and *n* = 6 for protein examination

Next, levels of free fatty acid (FFA) were examined to confirm PM_2.5_-induced lipid metabolic dysregulation in female mice. Appreciable accumulation in total FFA was detected in the livers of PM_2.5_ exposed female but not male animals as shown by Fig. 4a-c, Supplemental Fig. 4A-C. A closer look into individual FFAs revealed that unsaturated fatty acid of 16:1 increased in female mice exposed to PM_2.5_, albeit not reaching significant difference. Further, FFA levels of n-3 family or n-6 family (22:6, 22:5, 22:4, 20:5, 20:4, 18:3) but not n-9 family (20:3,18:1) displayed significant or a trend toward increase (Fig. 4c, Supplemental Fig. 4C). These observations suggest an enhancement of FFA levels in female mice induced by PM_2.5_ exposure.

To investigate the mechanism that serves to retain fatty acid in the liver, we examined molecules of fatty acid export, fatty acid uptake and TAG hydrolysis. Expression of ApoB, the molecules involved in TAG and fatty acid export, increased in PM_2.5_-exposed female mice at both mRNA and protein levels. However, no significant difference was observed with Apo E (Fig. 4d-h). Interestingly, PM_2.5_ exposure upregulated expression of ApoB at protein levels, but not mRNA levels in male mice (Fig. 4d, f and g, Supplemental Fig. 4D). Expression of microsomal triglyceride transort protein (MTTP), which produces beta-lipoprotein including ApoB, increased in response to PM_2.5_ exposure at both mRNA level and protein levels in female mice (Fig. 4e, f and h, Supplemental Fig. 4E). No significant difference in levels of MTTP expression was observed in male mice (Fig. 4d, f and g, Supplemental Fig. 4D). None of fatty acid-binding protein 1 (FABP1), FABP2, FABP5 or CD36, were altered at transcriptional levels in the liver of PM_2.5_-exposed mice, independent of sex factors (Fig. 4d and e). In addition, examination of molecules for lipolysis demonstrated that hepatic expression of both adipose triglyceride lipase (ATGL) and hormone-sensitive lipase (HSL) increased at both mRNA levels and protein levels in female, but not male, mice in response to PM_2.5_ exposure (Fig. 4d-h, Supplemental Fig. 4D-E). These results indicate that PM_2.5_ exposure enhanced hydrolysis of TAG and production of ApoB in the liver of female mice.

### Ambient PM_2.5_ exposure increased the Plasmalogens in the liver

**Fig. 5:**
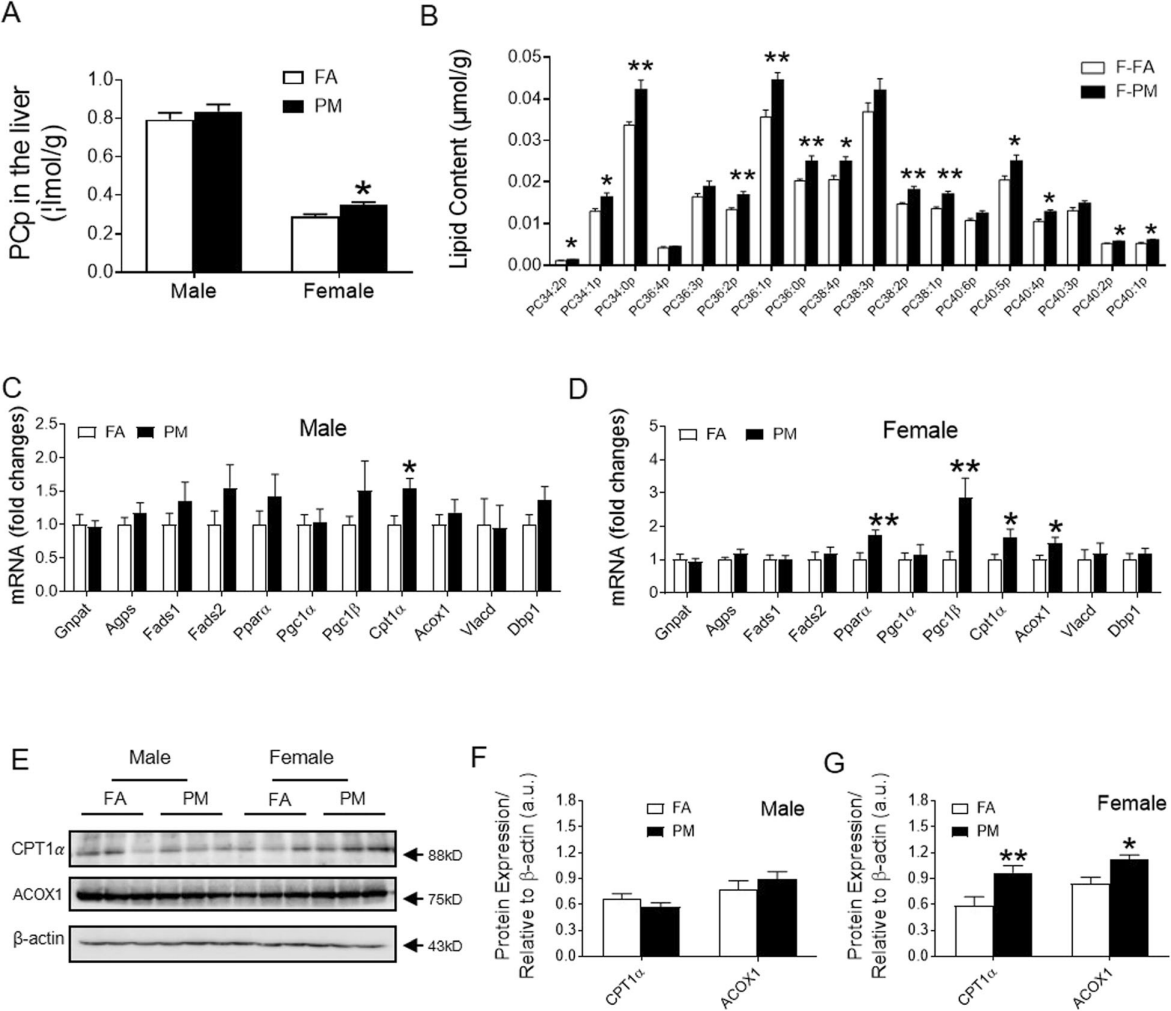
Effects of PM_2.5_ exposure on plasmalogen levels and relevant signals in the liver. **A**, Total plasmalogen content in the hepatic lipid extracts in male and female mice. **B**, Major plasmalogen species in the hepatic lipid extracts of female mice. **C** and **D**, mRNA levels of genes involved in plasmalogen synthesis, FADs and mitochondrial and peroxisomal FAO in the liver of male (C) and female (D) mice. **E**-**G**, Representative bands (E) and analyzed protein levels of CPT1αand ACOX1 in the liver of male (F) and female (G) mice. *P < 0.05, **P < 0.01 for each comparison. n = 5 for lipidomic analysis, n = 7–9 for mRNA examination, and n = 6 for protein examination

Plasmalogens are subclass of glycerophospholipid that contains vinyl ether and play multiple roles in cellular function by acting as components of the cell plasma membrane. Most plasmalogens have choline (PC-plasmalogens, PCp) in the polar head group and are enriched with n-3 or n-6 PUFAs, such as DHA (22:6 n-3) or arachidonic acid (20:4 n-6), in the sn-2 position. An investigation into the hepatic plasmalogens revealed increases in the levels of PCp in PM_2.5_ exposed female mice compared to their FA controls, while no such change was found in male mice exposed to PM_2.5_ (Fig. 5a, Supplemental Fig. 5A). Interestingly, detected PCp containing long chain and 0, 1, 2 or 5 double bonds (PC34:2p, PC34:1p, PC34:0p, PC36:2p, PC36:1p, PC36:0p, PC38:2p, PC38:1p, PC40:2p, PC40:1p, PC40:5p), PCp containing 4 double bonds (PC40:4p, PC38:4p, but not PC36:4p) in their structures significantly increased with PM_2.5_ exposure, whereas those with 3 double bonds (PC36:3p, PC38:3p, PC40:3p), were not altered (Fig. 5b, Supplemental Fig. 5B).

We next investigated potential reasons for increased plasmalogen levels. As shown in Fig. 5b-c, PM_2.5_ exposure showed no effect on hepatic mRNA expression of the rate-limiting enzymes in plasmalogen biosynthesis [glyceronephosphate O-acyltransferase (Gnpat) or alkylglycerone phosphate synthase (Agps)] or fatty acid desaturase (Fads1 and Fads2) which is associated with a decrease in DHA in hepatic lipids during plasmalogen synthesis either in male mice or female mice. Plasmalogens play a pivotal role in fatty acid metabolism in the liver and achieved this function by peroxisome proliferator-activated receptor alpha (PPARα). We found distinct increase in expression of PPARα and peroxisome proliferator-activated receptor gamma coactivator 1 beta (PGC1β) in liver from PM_2.5_-exposed female mice, whereas there was no significant difference in response to PM_2.5_ exposure in male mice (Fig. 5b and c). Carnitine palmitoyl transferase-1 (Cpt1α), acyl-CoA oxidase-1 (Acox1), very-long-chain acyl-CoA dehydrogenase (Vlcad), and D-bifunctional protein (Dbp1) are molecules implicated in mitochondrial and peroxisomal FAO. Consistent with changes in PPARα and PGC1β, Cpt1α and Acox1 were increased in expression at both mRNA levels (Fig. 5d) and protein levels (Fig. 5e and g, Supplemental Fig. 5C-D) in PM_2.5_-exposed female mice, while only Cpt1α increased at mRNA level in male mice (Fig. 5c).

### Ambient PM_2.5_ exposure inhibited HPA Axis

**Fig. 6:**
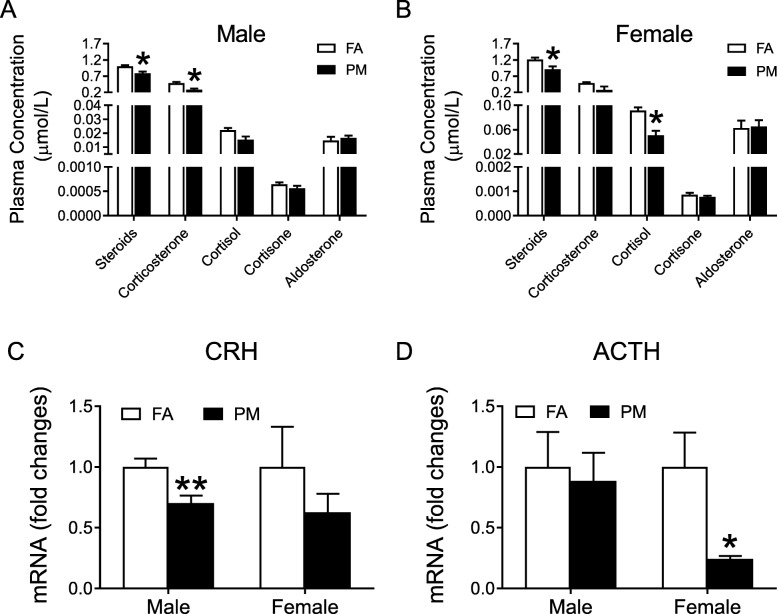
Effects of PM_2.5_ exposure on molecules of HPA axis. **A** and **B**, Levels of corticosteroids in plasma from male (A) and female (B) mice. **C** and **D**, mRNA levels of CRH (C) and ACTH (D) in the hypothalamus and pituitary gland of male and female mice. *P < 0.05, **P < 0.01 for each comparison. *n* = 4 for hormone analysis, n = 6 for mRNA examination

To explore the mechanism of PM_2.5_-induced metabolic dysfunction and female susceptibility, plasma hormones in relation to PM_2.5_ exposure were examined. Lower levels of steroids were observed in both male and female mice after PM_2.5_ exposure (Fig. 6a and b, Supplemental Fig. 6A-B). Level of corticosterone decreased in male mice whereas cortisol decreased in female mice (Fig. 6a and b, Supplemental Fig. 6A-B). Next, mRNA levels of corticotropin-releasing hormone (CRH) in hypothalamus decreased in male mice (Fig. 6c) and adrenocorticotropic hormore (ACTH) in pituitary decreased in female mice (Fig. 6d) in response to PM_2.5_ challenge. In addition, circulating sex hormones were also examined with mice exposed to PM_2.5_. As shown in Supplemental Fig. 6C-F, PM_2.5_ significantly increased testosterone levels in the plasma in male mice (Supplemental Fig. 6C, E). There was no significant alteration in other hormones observed (Supplemental Fig. 6C-F).

## Discussion

The present study demonstrated greater susceptibility in IR and hepatic lipid accumulation in response to PM_2.5_ exposure in female mice than male mice. It provides a readout of lipid metabolites in response to continuous real-world PM_2.5_ exposure, including increase in levels of hepatic lipids and cholesterol, and dysregulation of plasmalogens. Furthermore, PM_2.5_ exposure inhibited HPA gland axis and glucocorticoids levels, which may contribute to the sex difference in PM_2.5_-induced metabolic dysfunction.

We found distinct changes in the levels of TAG in the liver in response to continuous ambient PM_2.5_ exposures. Previously, we have observed PM_2.5_-induced hepatic steatosis under both normal chow and high fat diet situations [5, 6]. Enhanced lipogenesis has been demonstrated to account for triglyceride deposition in the liver in PM_2.5_ exposed mice [5, 8]. Intracellular concentration of fatty acid and its structure are useful indicators of lipid metabolism, which is essential to understand the molecular mechanisms underlying the metabolic syndrome. Accumulation of C16:0, C16:1, C18:0 and C18:1 mostly suggests the enhanced biosynthesis in response to pathophysiological stimulation such as metabolic disorder [15]. In addition to enhancement of FA synthesis [5], increased levels of fatty acid may be also derived from either excessive uptake of extracellular FA, reduced oxidation of these fatty acids or increased lipohydrolysis. Non-significant changes in genes encoding fatty acid uptake excluded the excess uptake of fatty acids. Enhancement of CPT1α and ACOX1 (two enzymes for FAO) was against the inhibition of FAO too. Whereas, increased expression of HSL and ATGL was observed, indicating enhancement of lipolysis which leads to increased fatty acid as products. Thus, the increased fatty acid production and lipolysis contributed to the elevation of fatty acids, which may induce compensatory adaptation of FAO.

Apolipoprotein B is a structural protein and constitutes the integral component of chylomicrons, very low density lipoprotein (VLDL), intermediate density lipoprotein and low density lipoprotein particles as well. VLDL, which is secreted by the liver, contains apolipoprotein B100 (apoB100) and transports triglycerides. Similar to the IR state, the increased flux of FFA promotes hepatic TG production, which subsequently induces apoB synthesis and secretion [16]. As ApoB is synthesized, it is lipidated by MTTP. Then triglycerides are added to the nascent ApoB by MTTP, producing a dense, lipid-poor, pre-VLDL particle and exporting to extrahepatic tissues to balance the lipid level in the liver. Consistent with it, apoB and/or MTTP were highly expressed in PM-exposed mice, which may be triggered by increased hepatic TG. Evidence has been provided for a molecular link between hepatic apoB100 and attenuated insulin signaling [17]. Thus, the significant increase in apoB in mice may contribute to the attenuated insulin sensitivity.

A novel finding is the highly significant increase in PC-plasmalogen in PM_2.5_ exposed mice relative to FA-exposed mice. Plasmalogens are a crucial subclass of glycerophospholipid that contains vinyl ether and regulate multiple cellular functions as important components of the cell plasma membrane [18]. Decrease in plasmalogen levels have been shown to be related to metabolic diseases [19, 20]. Jang et al. demonstrated that the hepatic plasmalogens protect against hepatic steatosis and steatohepatitis through PPARα-dependent activation of FAO in mice [21]. Although attenuated insulin sensitivity and enhanced lipid accumulation in the liver was observed in the PM_2.5_-exposed mice, increased plasmalogens were shown as well. Consistent with the increased level of plasmalogens, we observed elevated expression of PPARα, a nuclear receptor that stimulates transcription of mitochondrial and peroxisomal FAO in the liver [22, 23], followed by upregulation of CPT1α (a hepatic mitochondrial FAO) and ACOX1 (a peroxisomal FAO). The explanation for the simultaneous presence of increased plasmalogens and lipid accumulation is that the mice in our study were exposed as long as 6 months, which may induce self-adaptation to upregulate the synthesis of plasmalogens to confront PM_2.5_-induced damage. However, the exact mechanism and time-window of compensatory adaptation await further investigation.

Another important finding was the sex difference in response to PM_2.5_ exposure. Glucocorticoids work as stress hormone in response to adverse stimulation. Results from this study demonstrated significant decrease in plasma levels of steroids, cortisol or corticosterone in male and/or female mice in response to PM_2.5_ exposure. Glucocorticoids are the marker of HPA axis and regulated by ACTH from pituary and CRH from hypothalamus. In line with it, expression levels in both CRH and ACTH were inhibited in PM-inhaled male and female mice respectively suggesting inhibition of HPA axis by long-term PM_2.5_ exposure. This is contrary to a clinical trial of 9-day [24] and an animal study with acute PM exposure (within 4 days) [25] which demonstrated increase in glucocorticoids in the group with higher levels of PM_2.5_ exposure. It is possible that short term PM_2.5_ inhalation induces CRH release from hypothalamus, which stimulates the anterior pituitary gland to release ACTH into circulation and then targets the adrenal cortex to synthesize and release glucocorticoids to react the adverse environmental stimulus. What should be kept in mind is that synthesis and secretion of glucocorticoids were autoregulated by negative feedback cycles and the HPA axis may be dysregulated with stress. Studies have shown that exposure to high GC levels (stress) suppressed the HPA response to hypoglycemia stimulation [26], which were significantly reduced in diabetic animals compared with controls as well [27]. Thus, hypofunction of HPA axis may be induced by the negative feedback of autoregulation after long-term (6 months) PM_2.5_ exposure or IR situation in the present study. Considering the important role of glucocorticoids in maintaining the resting and stress-related homeostasis as a stress hormone, the hypofunction of HPA axis and decreased glucocorticoids in turn may contribute to the vulnerability of IR and hepatic lipid accumulation to PM_2.5_ challenge.

A potential limitation of the study is the failure to examine the PM_2.5_’s effects at different time windows. A presentation of molecules for lipohydrolysis enzymes, plasmalogen and its downstream signals with short and long term PM_2.5_ exposure duration would verify and elucidate the adaptive response with stronger evidence. However, this concern could be mitigated by our further exploration into the self-compensatory regulation. Secondly, no effect was found on lung inflammation after PM_2.5_ exposure in both sex (data not shown). However, as breathing activity between male and female mice differs, the contribution of discrepant deposited dose of PM in the lung to the observed effects was unknown. Thirdly, we could not clarify the exact metabolic signaling targets by which glucocorticoids and mineralocorticoids regulate and induce sex difference with the present work. In addition, although no PM_2.5_ effect on sex hormones was observed, whether the downstream of sex hormones was disturbed was not examined. Fourthly, catecholamines, which are released from the medullary layer of the adrenal gland, are stimulator of not only HPA axis but also lipolysis. Examination of catecholamines could help to clarify the mechanism by which PM_2.5_ exposure elevated hepatic levels of lipase expression in female mice. Finally, Since whole body exposure leads to particle uptake by both inhalation and oral ingestion (licking of pelt), whether more pronounced liver damage in female mice due to higher oral uptake (more mutual grooming in female mice) need to be verified by PM_2.5_ mass quantification in organs.

## Conclusions

In summary, this is the first-ever animal study to demonstrate sex differences in insulin sensitivity and hepatic lipid accumulation after “real world” exposure to PM_2.5_. The present study suggests significant changes in HPA axis and changes in lipid metabolites. Our novel findings provide insights into the potential mechanisms of the adverse metabolic health effects in sex difference. More research regarding sex-dimorphic pathophysiological mechanisms of PM’s adverse effects could contribute to more personalized care in the future and would thus promote awareness in terms of sex-specific risk factors.

## Methods

### Animal care and use

C57BL/6 mice of male and female at 10-week-old were purchased from Shanghai Laboratory Animal Co., Ltd. (SLAC, Shanghai, China). All mice were maintained at 21 °C on a 12-h light/12-h dark cycle with free access to water and diet (Xietong Organism, Nanjing, China). Animal experiments were accordant with the National Institute of Health Guide for the Care and Use of Laboratory Animals. The Animal Care and Use Committee at Zhejiang Chinese Medical University approved the experimental protocals.

### Ambient whole-body inhalational protocol

Both male and female C57BL/6 mice were continuously whole body exposed by inhalation to either filtered air (FA) or PM_2.5_ (PM) from ambient air from November 4, 2016 to April 20, 2017, for a total duration of ~ 24 weeks, in a set of exposure system (“ZheJiang Whole-body Exposure System 1 (ZJ-WES1)” located at Zhejiang Chinese Medical University in Hangzhou). ZJ-WES1 is a versatile whole body aerosol system which allows us to perform the studies on animal models that recapitulate true personal, long-term exposure to the direct environmental PM_2.5_. The system includes two temperature-controlled chambers. The PM chamber is connected to the ambient air with particulate matters larger than 2.5 μm removed using a cyclone and particles evenly distributed in the chamber. The FA chamber is equipped with an HEPA filters positioned in the inlet valve to remove all of the PM_2.5_ in the air stream. Male and female mice were housed in separated cages with 4–5 mice/cage in each chamber. Monitoring of the FA and PM exposure environment within the two chambers was continuously recorded with an Aerosol Monitoring Meter (Thermal Scientific, China), which being zeroed with the zero-adjusting device according to the manual before usage. The data of ambient aerosol concentration were collected from the local monitoring station, which was located at the cross of Jiangnan Ave. and Jiangling Rd., 10 km away from the study site.

### PM_2.5_ concentration and composition in the exposure chamber

To analyze the major elements of particles, PM_2.5_ samples in the exposure chambers were collected on Teflon filter membranes (37 mm in diameter with 2 μm pore; GE healthcare, Amersham Place, UK). The membranes were weighed in a temperature- and humidity-controlled weighing room using a Excellence Plus XP microbalance (Mettler Toledo, Schweiz) before and after sampling. Weight gains were used to calculate the exposed concentrations. Analyses for major elements (Sb, Al, Se, Cd, Pb, Ni, Tl, Mn, Ca, Na, K, Fe and Zn) were performed using ICP-MS (Thermo Fisher Scientific, Bremen, Germany) by Pooke Testing company at Hangzhou.

### Measurements of blood glucose homeostasis and insulin sensitivity

At the end of the exposure to FA or PM_2.5_, mice were fasted overnight and underwent assessment of fasting insulin/glucose levels and intraperitoneal glucose tolerance test (IPGTT) with dextrose (2 mg/g body weight) injected intra-peritoneally. Blood glucose was measured with a FreeStyle Blood Glucose Meter (Abbott Diabetes Care Inc., Alameda, CA) before and at 30, 60, 90, and 120 min after dextrose injection. Fasting insulin levels in serum were examined with Mouse Ultrasensitive Insulin ELISA (Crystal Chem, Elk Grove Village, IL, USA). Based on 1 mg of insulin as equivalent to 24 IU, HOMA-IR was calculated according to the formula HOMA = [fasting insulin concentration (ng/mL) × 24 × fasting glucose concentration (mg/dL)]/405. ITT was applied to measure insulin sensitivity in mice fasted for 4.5 h. human regular insulin (0.5 U/kg) (Lilly, France) was administered by intra-peritoneal injection. Then, blood glucose was measured at the same time points as IPGTT.

### Tissue collection

At the end of the PM_2.5_ exposure, blood was collected from the fundus venous plexus after mice inhalation of overdose CO_2_ and then mice were perfused transcardially with ice-cold 0.1 *M* PBS. Brains were removed and hypothalamus were collected and frozen in liquid nitrogen for further investigation. Livers were removed immediately and then fixed by 10% neutral Formalin or frozen for further usage. To minimize the effect of circadian rhythm on results for HPA axis and liver organ, tissues were collected only in the morning, with the alternative sequence of FA and PM.

### Oil red O staining

Segments of liver were embedded in Optimal Cutting Temperature compound (Tissue-Tek, Sakura Finetek USA Inc., Torrance, Calif) for oil red-O staining.

### Immunoblotting

Liver tissue were homogenized with M-PER Mammalian protein extraction reagent (Thermo Scientific), loaded on 10% SDS-PAGE gel and transferred to immobilon-P polyvinylidenedifluoride membranes and incubated with different primary antibodies. After washing, the membranes were then followed by incubation with a secondary antibody conjugated with horseradish peroxidase. Finally, the immunoblots were visualized with chemiluminescence imaging system (Bio-Rad, USA) and analyzed with ImageJ software. β-actin was used as control reference.

### Quantitative RT-PCR

RT-PCR was performed using RNA extracted from tissue of liver, hypothalamus and pituitary gland from the experimental mice. Expression levels of molecules were calculated using the ΔCt method relative to β-actin and expressed as relative mRNA levels compared with FA control. The sequences of all primers are list in Supplemental Material, Table S3.

### Lipid extraction

The details of the extraction protocol have been previously described elsewhere [28]. Briefly, frozen liver tissues were firstly inactivated chloroform:methanol (1:2) with 10% deionized H_2_O (900 μL). Then samples were homogenized on an automated bead ruptor (Omni, USA) and further centrifuged at 1500 rpm, 4 °C, 1 h. Next, 400 μL of deionized H_2_O and 300 μL of chloroform were added to break phase. After transferring the lower organic phase into another tube, 500 μL chloroform was added for a second extraction. The two extractions were pooled together and dried using SpeedVac (Genevac, UK). The dried samples were stored at − 80 °C for mass spectrometric analysis.

### Lipidomics analysis

LC/MS analyses were carried out on an Exion UPLC coupled with Sciex QTRAP 6500 Plus. Peaks with desirable shapes and signal-to-noise ratios more than 3 were identified.

Analysis of phospholipids and sphingolipids. The analytical protocol has been described in details previously [28, 29]. A Phenomenex Luna 3 μ silica column (i.d. 150 × 2.0 mm) was used to separate individual lipid classes of polar lipids by normal phase HPLC. In brief, chloroform:methanol:ammonium hydroxide (89.5:10:0.5), and chloroform:methanol:ammoniumhydroxide:water (55:39:0.5:5.5) were used as mobile phase A and B, respectively. Polar lipids were quantified by multiple reaction monitoring transitions. Spiked internal standards obtained from Avanti Polar Lipids (Alabaster, AL, USA) and LIPIDS MAPS were referred for quantification of individual lipid species. GM3-d18:1/17:0 was synthesized in-house. Prior to sample analysis, the exact amounts of all standards were pre-corrected against quantitative standards. All the LC-MS analyses were conducted according to the criteria that only peaks reaching the limit of quantitation and falling within the linearity range were analyzed for further quantitation.

Analysis of neutral lipids. Reverse phase HPLC/ESI/MS/MS with modification was performed to analyze neutral lipids (TAGs, DAGs and CEs). The analysis has been described previously [30, 31]. Briefly, a PhenomenexKinetex 2.6 μ-C18 column (i.d. 4.6 × 100 mm) was applied to separate the neutral lipids lipids aforementioned. The isocratic mobile phase was set as chloroform:methanol:0.1 M ammonium acetate at the ratio of 100:100:4, at flow rate of 150 μL/min, 22 min. The levels of TAG were calculated as relative contents to the spiked d5-TAG 42:0, d5-TAG 48:0, d5-TAG 54:0 internal standards (CDN isotopes), while DAG species and CE species were quantified using d5-DAG (18:1/18:1), d5-DAG (16:0/16:0) (Avanti Polar Lipids, Alabaster, AL, USA) and d6-CE (CDN isotopes) as internal standards, respectively.

Analysis of free cholesterol. HPLC/APCI/MS/MS was conducted for free cholesterols analysis with d6-Cho (CDN isotopes) as internal standard [32].

### Hormone analysis

Steroids were extracted from 100 μL of serum with methyl-tert butyl ether and clean supernatant was collected. The extraction was repeated once and the extracts were pooled and dried in the SpeedVac (GeneVac, UK). Dried extract was resuspended in methanol:water (1:1) containing d_4_-estrone, d_3_-testosterone, d_9_-progesterone, d_4_-estradiol, d_4_-pregnenolone. LCMS analyses of steroids were conducted on a Thermofisher DGLC coupled to Sciex QTRAP 6500 Plus. Individual steroids were quantitated by normalizing to intensities of spiked internal standards.

### Data analysis

Data are expressed as means ± standard error of the mean (SEM) and presented in two formats of absolute values and normalization relative to its FA respectively. Graphpad Prism software (Version 6) was used for 2-tailed Student’s t test when comparing PM with FA. Two-way ANOVA followed by bonferroni’s multiple comparisons test was applied to distinguish the effects of PM exposure from the effects of sex. *P* value of < 0.05 was considered statistically significant. Principal component analysis was performed to summarize and visualize high-dimensional lipidomics data. Individual sample was indicated by point over the first two principal components, which explain xx% of total variance in the data. Concentration ellipses were added to visualize group distribution, assuming multivariate normal distribution. Mann-Whitney-U test P value, and fold change of each lipid were illustrated in the volcano plots. Lipids with *P* < 0.05 indicated in red, and metabolites with top 10 smallest *P* values were labeled by their names. Statistical analyses were performed using R 3.5.

## Supplementary information

**Additional file 1: Supplemental Materials. Supplemental Figure 1.** Body weight and fasting blood glucose before PM_2.5_ exposure in C57BL/6 mice. **A**, Absolute value of body weight. **B**, Absolute value of fasting blood glucose. **C**, Fold change of body weight relative to FA. **D**, Fold change of blood glucose relative to FA. n=8. **Supplemental Figure 2.** Effects of PM_2.5_ exposure on body weight and glucose homeostasis in C57BL/6 mice at the end of PM_2.5_ exposure. **A,** Fold change of body weight relative to FA. **B,** Fold change of fasting blood glucose relative to FA. **C-D,** Fold change of fasting insulin levels (C) and HOMA-IR (D). **E,** Fold change of AUC (area under curves) of ITT relative to FA. *P<0.05, **P<0.01 for each comparison. n=5-8. **Supplemental Figure 3.** Effects of PM_2.5_ exposure on neutral lipids and cholesterol ester (CE) in the liver. **A,** Fold change of total DAG levels relative to FA. **B,** Fold change of total TAG levels relative to FA. **C,** Fold change of total cholesteroal levels relative to FA. **D,** Levels of total CE levels. **E-F,** levels in CE species in male (E) and female (F) mice. **G,** Fold change of total CE levels relative to FA. **H-I,** Fold change of levels in CE species relative to FA in male (H) and female (I) mice.*P<0.05 for each comparison. n=5. **Supplemental Figure 4.** Effects of PM_2.5_ exposure on FFA profile and relevant signals in the liver. **A,** Fold change of total FFA content relative to FA in the hepatic lipid extracts from mice. **B and C,** Fold change of major species of FFA relative to FA in the hepatic lipid extracts from male (B) and female (C) mice. **D and E,** Fold change of analyzed protein levels of ApoB, MTTP, ATGL and HSL relative to FA in liver tissue from male (D) and female (E) mice. *P<0.05 for each comparison. n=5 for lipidomic analysis and n=6 for protein examination. **Supplemental Figure 5.** Effects of PM_2.5_ exposure on plasmalogen levels and relevant signals in the liver. **A,** Fold change of total plasmalogen content relative to FA in the hepatic lipid extracts in mice. **B**, Fold change of major plasmalogen species relative to FA in the hepatic lipid extracts of female mice. **C and D,** Fold change of analyzed protein levels of CPT1αand ACOX1 relative to FA in the liver of male (C) and female (D) mice. *P<0.05, **P<0.01 for each comparison. n=5 for lipidomic analysis and n=6 for protein examination. **Supplemental Figure 6.** Effects of PM_2.5_ exposure on corticosteroids and sex hormones in mice. **A and B,** Fold change in levels of corticosteroids relative to FA in plasma from male (A) and female (B) mice. **C and D,** Levels of sex hormones in plasma from male (C) and female (D) mice. **E and F,** Fold change in levels of sex hormones relative to FA in plasma from male (E) and female (F) mice. *P<0.05 for each comparison. n=4. **Supplemental Table 1.** Elemental constituents of the exposed PM_2.5_.

## Data Availability

Not applicable.

## Abbreviations

Acox1: Acyl-CoA oxidase-1
ACTH: Adrenocorticotropic hormore
AGPS: Alkylglycerone phosphate synthase
ATGL: Adipose triglyceride lipase
CE: Cholesterol ester
CPT1: Carnitine palmitoyl transferase 1
CRH: Corticotropin-releasing hormone
DAG: Diacylglycerols
DBP1: D-bifunctional protein 1
FA: Filtered air
FABP: Fatty acid-binding protein
FAO: Fatty acid oxidation
Fads: Fatty acid desaturase
FFA: Free fatty acid
Gnpat: Glyceronephosphate O-acyltransferase
HOMA-IR: Homeostasis Model Assessment of Insulin Resistance
HPA: Hypothalamus-pituitary-adrenal
HSL: Hormone-sensitive lipase
IPGTT: Intraperitoneal glucose tolerance test
IR: Insulin resistance
ITT: Insulin tolerance test
MTTP: Microsomal triglyceride transport protein
MUFA: Monounsaturated fatty acid
PCA: Principal component analysis
PCp: PC-plasmalogen
PGC1β: Peroxisome proliferator-activated receptor gamma coactivator 1 beta
PM_2.5_: Particulate matter ≤2.5 μm, fine particulate matter
PPARα: Peroxisome proliferator-activated receptor alpha
PUFA: Polyunsaturated fatty acid
SFA: Saturated fatty acid
TAG: Triacylglycerols
VLCAD: Very-long-chain acyl-CoA dehydrogenase
VLDL: Very low density lipoprotein
ZJ-WES1: ZheJiang Whole-body Exposure System 1
