# Supplementary material for: Sex-dependent effects of ambient PM2.5 pollution on insulin sensitivity and hepatic lipid metabolism in mice
Source: Part Fibre Toxicol. 2020 Apr 22;17:14. doi: 10.1186/s12989-020-00343-5 (PMC7178763; doi:10.1186/s12989-020-00343-5)
Supplement: Supplementary file 1 — Additional file 1: Supplemental Materials. Supplemental Figure 1. Body weight and fasting blood glucose before PM2.5 exposure in C57BL/6 mice. A, Absolute value of body weight. B, Absolute value of fasting blood glucose. C, Fold change of body weight relative to FA. D, Fold change of blood glucose relative to FA. n=8. Supplemental Figure 2. Effects of PM2.5 exposure on body weight and glucose homeostasis in C57BL/6 mice at the end of PM2.5 exposure. A, Fold change of body weight relative to FA. B, Fold change of fasting blood glucose relative to FA. C-D, Fold change of fasting insulin levels (C) and HOMA-IR (D). E, Fold change of AUC (area under curves) of ITT relative to FA. *P<0.05, **P<0.01 for each comparison. n=5-8. Supplemental Figure 3. Effects of PM2.5 exposure on neutral lipids and cholesterol ester (CE) in the liver. A, Fold change of total DAG levels relative to FA. B, Fold change of total TAG levels relative to FA. C, Fold change of total cholesteroal levels relative to FA. D, Levels of total CE levels. E-F, levels in CE species in male (E) and female (F) mice. G, Fold change of total CE levels relative to FA. H-I, Fold change of levels in CE species relative to FA in male (H) and female (I) mice.*P<0.05 for each comparison. n=5. Supplemental Figure 4. Effects of PM2.5 exposure on FFA profile and relevant signals in the liver. A, Fold change of total FFA content relative to FA in the hepatic lipid extracts from mice. B and C, Fold change of major species of FFA relative to FA in the hepatic lipid extracts from male (B) and female (C) mice. D and E, Fold change of analyzed protein levels of ApoB, MTTP, ATGL and HSL relative to FA in liver tissue from male (D) and female (E) mice. *P<0.05 for each comparison. n=5 for lipidomic analysis and n=6 for protein examination. Supplemental Figure 5. Effects of PM2.5 exposure on plasmalogen levels and relevant signals in the liver. A, Fold change of total plasmalogen content relative to FA in the hepatic lipid extra [file 12989_2020_343_MOESM1_ESM.docx]

**Supplemental Material**

**Sex-dependent effects of ambient PM_2.5_ pollution on insulin sensitivity and hepatic lipid metabolism in mice**

Ran Li ^1,2#^, Qing Sun^1,2#^, Sin Man Lam^3^, Rucheng Chen^1,2^, Junyao Zhu^1^, Weijia Gu^1,2^, Lu Zhang^1,2^, He Tian^3^, Kezhong Zhang^4^, Lung-Chi Chen^5^, Qinghua Sun^6^, Guanghou Shui^3^*, Cuiqing Liu^1,2^*

**Contents of Supplemental Material**

Supplemental Figure 1

Supplemental Figure 2

Supplemental Figure 3

Supplemental Figure 4

Supplemental Figure 5

Supplemental Figure 6

Supplemental Table 1

Supplemental Table 1

Supplemental Table 1

**Supplemental Figure 1.** Body weight and fasting blood glucose before PM_2.5_ exposure in C57BL/6 mice. A, Absolute value of body weight. B, Absolute value of fasting blood glucose. C, Fold change of body weight relative to FA. D, Fold change of blood glucose relative to FA. n=8.

**Supplemental Figure 2. Effects of PM_2.5_ exposure on body weight and glucose homeostasis in C57BL/6 mice** **at the end of PM_2.5_ exposure. A,** Fold change of body weight relative to FA. **B,** Fold change of fasting blood glucose relative to FA. **C-D,** Fold change of fasting insulin levels (C) and HOMA-IR (D). **E,** Fold change of AUC (area under curves) of ITT relative to FA. **P*<0.05, ***P*<0.01 for each comparison. n=5-8.


**Supplemental Figure 3. Effects of PM_2.5_ exposure on neutral lipids and cholesterol ester (CE) in the liver**. **A,** Fold change of total DAG levels relative to FA. **B,** Fold change of total TAG levels relative to FA. **C,** Fold change of total cholesteroal levels relative to FA. **D,** Levels of total CE levels. **E-F,** levels in CE species in male (E) and female (F) mice. **G,** Fold change of total CE levels relative to FA. **H-I,** Fold change of levels in CE species relative to FA in male (H) and female (I) mice.**P*<0.05 for each comparison. n=5.

**Supplemental Figure 4. Effects of PM_2.5_ exposure on FFA profile and relevant signals in the liver**. **A,** Fold change of total FFA content relative to FA in the hepatic lipid extracts from mice. **B and C,** Fold change of major species of FFA relative to FA in the hepatic lipid extracts from male (B) and female (C) mice. **D and E,** Fold change of analyzed protein levels of ApoB, MTTP, ATGL and HSL relative to FA in liver tissue from male (D) and female (E) mice. **P*<0.05 for each comparison. n=5 for lipidomic analysis and n=6 for protein examination.

**Supplemental Figure 5. Effects of PM_2.5_ exposure on plasmalogen levels and relevant signals in the liver**. **A,** Fold change of total plasmalogen content relative to FA in the hepatic lipid extracts in mice. **B**, Fold change of major plasmalogen species relative to FA in the hepatic lipid extracts of female mice. **C and D,** Fold change of analyzed protein levels of CPT1αand ACOX1 relative to FA in the liver of male (C) and female (D) mice. **P*<0.05, ***P*<0.01 for each comparison. n=5 for lipidomic analysis and n=6 for protein examination.

**Supplemental Figure 6. Effects of PM_2.5_ exposure on corticosteroids and sex hormones in mice**. **A and B,** Fold change in levels of corticosteroids relative to FA in plasma from male (A) and female (B) mice. **C and D,** Levels of sex hormones in plasma from male (C) and female (D) mice. **E and F,** Fold change in levels of sex hormones relative to FA in plasma from male (E) and female (F) mice. **P*<0.05 for each comparison. n=4.

**Supplemental Table 1. Elemental constituents of the exposed PM_2.5_**

| Elements | PM_2.5_, ng/mg | |
| --- | --- | --- |
|  | Mean | SD |
| Ca | 11699.0 | 6662.8 |
| K | 7024.8 | 4154.0 |
| Fe | 6373.5 | 3956.9 |
| Al | 3136.1 | 1747.5 |
| Zn | 2829.1 | 1690.0 |
| Pb | 804.4 | 479.7 |
| Mn | 513.8 | 366.0 |
| Cd | 86.1 | 126.2 |
| Sb | 73.1 | 77.2 |
| Se | 52.5 | 43.3 |
| Tl | 5.5 | 4.3 |
| Na | UDL |  |

n= 10 filters. UDL: under detection limit

**Supplemental Table 2. P values for comparison of TAG species in liver from mice exposed to PM_2.5_**

| TAG Species | P values | | |  | TAG Species | P values | | | | |
| --- | --- | --- | --- | --- | --- | --- | --- | --- | --- | --- |
|  | M-PM vs  M-FA | F-PM vs  F-FA | F-PM vs M-PM |  |  | M-PM vs  M-FA | F-PM vs  F-FA | | F-PM vs M-PM | |
| TAG50:3(16:0) | 0.03^*^ | 0.02^*^ | 0.10 |  | TAG50:3(16:2) | 0.15 | 0.03^*^ | 0.02^*^ | |  |
| TAG50:2(16:0) | 0.22 | 0.03^*^ | 0.31 |  | TAG48:3(18:2) | 0.31 | 0.01^*^ | 0.01^*^ | |  |
| TAG52:4(16:0) | 0.03^*^ | 0.02^*^ | 0.01^*^ |  | TAG48:2(18:2) | 0.31 | 0.10 | 0.01^*^ | |  |
| TAG52:3(16:0) | 0.01^*^ | 0.02^*^ | 0.01^*^ |  | TAG50:4(18:2) | 0.22 | 0.01^*^ | 0.01^*^ | |  |
| TAG52:2(16:0) | 0.42 | 0.06 | 0.02^*^ |  | TAG50:3(18:2) | 0.03^*^ | 0.01^*^ | 0.03^*^ | |  |
| TAG54:5(16:0) | 0.06 | 0.06 | 0.01^*^ |  | TAG52:4(18:2) | 0.03^*^ | 0.02^*^ | 0.01^*^ | |  |
| TAG54:4(18:0) | 0.55 | 0.42 | 0.01^*^ |  | TAG52:3(18:2) | 0.01^*^ | 0.06 | 0.01^*^ | |  |
| TAG54:2(18:0) | 0.84 | 0.42 | 0.01^*^ |  | TAG52:2(18:2) | 0.15 | 0.06 | 0.01^*^ | |  |
| TAG54:1(18:0) | 0.15 | 0.84 | 0.03^*^ |  | TAG54:7(18:2) | 0.15 | 0.03^*^ | 0.01^*^ | |  |
|  |  |  |  |  | TAG54:6(18:2) | 0.10 | 0.06 | 0.01^*^ | |  |
| TAG50:3(16:1) | 0.15 | 0.01^*^ | 0.42 |  | TAG54:5(18:2) | 0.10 | 0.22 | 0.01^*^ | |  |
| TAG52:4(16:1) | 0.15 | 0.03^*^ | 0.03^*^ |  | TAG54:4(18:2) | 0.22 | 0.10 | 0.01^*^ | |  |
| TAG52:3(16:1) | 0.42 | 0.10 | 0.03^*^ |  | TAG54:3(18:2) | 0.10 | 0.84 | 0.01^*^ | |  |
| TAG54:4(16:1) | 0.15 | 0.03^*^ | 1.00 |  | TAG56:8(18:2) | 0.15 | 0.01^*^ | 0.01^*^ | |  |
| TAG48:2(18:1) | 0.02 | 0.22 | 0.22 |  | TAG56:7(18:2) | 0.03^*^ | 0.06 | 0.01^*^ | |  |
| TAG48:1(18:1) | 0.69 | 0.42 | 0.02^*^ |  | TAG56:6(18:2) | 0.06 | 0.15 | 0.01^*^ | |  |
| TAG50:3(18:1) | 0.42 | 0.01^*^ | 0.03^*^ |  | TAG56:3(18:2) | 0.55 | 0.31 | 0.03^*^ | |  |
| TAG52:4(18:1) | 0.15 | 0.06 | 0.03^*^ |  | TAG56:5(20:2) | 0.42 | 0.22 | 0.01^*^ | |  |
| TAG52:3(18:1) | 0.03^*^ | 0.06 | 0.02^*^ |  | TAG56:4(20:2) | 0.55 | 0.55 | 0.02^*^ | |  |
| TAG52:2(18:1) | 0.42 | 0.06 | 0.02^*^ |  |  |  |  |  | |  |
| TAG52:1(18:1) | 0.69 | 0.42 | 0.02^*^ |  | TAG52:4(18:3) | 0.03^*^ | 0.02^*^ | 0.01^*^ | |  |
| TAG54:5(18:1) | 0.15 | 0.22 | 0.01^*^ |  | TAG54:7(18:3) | 0.15 | 0.06 | 0.01^*^ | |  |
| TAG54:4(18:1) | 0.31 | 0.10 | 0.01^*^ |  | TAG54:6(18:3) | 0.15 | 0.15 | 0.01^*^ | |  |
| TAG54:3(18:1) | 0.84 | 0.42 | 0.01^*^ |  | TAG54:5(18:3) | 0.31 | 0.15 | 0.01^*^ | |  |
| TAG54:2(18:1) | 0.69 | 1.00 | 0.01^*^ |  | TAG54:4(18:3) | 0.10 | 0.69 | 0.01^*^ | |  |
| TAG56:8(18:1) | 1.00 | 0.22 | 0.01^*^ |  | TAG56:6(20:3) | 0.42 | 0.42 | 0.01^*^ | |  |
| TAG56:5(18:1) | 0.06 | 0.10 | 0.01^*^ |  | TAG56:5(20:3) | 0.31 | 0.31 | 0.01^*^ | |  |
|  |  |  |  |  | TAG56:7(20:5) | 0.69 | 0.02^*^ | 0.01^*^ | |  |
| TAG54:7(20:4) | 0.10 | 0.03^*^ | 0.01^*^ |  | TAG56:7(22:5) | 0.01^*^ | 0.01 | 0.01^*^ | |  |
| TAG54:6(20:4) | 0.01^*^ | 0.03^*^ | 0.01^*^ |  | TAG56:6(22:5) | 0.06 | 0.03^*^ | 0.01^*^ | |  |
| TAG54:5(20:4) | 0.15 | 0.31 | 0.01^*^ |  | TAG58:8(22:5) | 0.69 | 0.06 | 0.01^*^ | |  |
| TAG56:7(20:4) | 0.22 | 0.31 | 0.01^*^ |  | TAG54:7(22:6) | 0.01^*^ | 0.01^*^ | 0.01^*^ | |  |
| TAG56:6(20:4) | 0.10 | 0.84 | 0.01^*^ |  | TAG56:8(22:6) | 0.01^*^ | 0.01^*^ | 0.01^*^ | |  |
| TAG58:8(20:4) | 0.42 | 0.31 | 0.01^*^ |  | TAG56:7(22:6) | 0.06 | 0.01^*^ | 0.01^*^ | |  |
| TAG56:6(22:4) | 0.01^*^ | 0.15 | 0.02^*^ |  | TAG58:9(22:6) | 0.06 | 0.02^*^ | 0.01^*^ | |  |
| TAG56:5(22:4) | 0.42 | 0.03^*^ | 0.01^*^ |  | TAG58:8(22:6) | 0.15 | 0.03^*^ | 0.01^*^ | |  |

TAG species in the liver were measured by lipidomics after 6-month PM_2.5_ exposure. n=5.

**Supplemental Table 3. Primers used for real-time PCR**

| Genes | Forward primer | Reverse primer |
| --- | --- | --- |
| *Apob* | AAACATGCAGAGCTACTTTGGAG | TTTAGGATCACTTCCTGGTCAAA |
| *Apoe* | GAGGAACAGACCCAGCAAATA | TTGTTGCAGGACAGGAGAAG |
| *Mttp* | GCTTCCGTTAAAGGTCACACA | TTTGTAGCCCACGCTGTCTT |
| *Fabp1* | TCAAGCTGGAAGGTGACAATAA | GTCTCCATTGAGTTCAGTCACG |
| *Fabp2* | TCGGTTCCTGAGGATACAAGAT | TTTGATGACTGTGGGATTGAAG |
| *Fabp5* | ACAGGGTTTTTGCATTCCTG | TTGGTTCTTTCGAACCTTG |
| *CD36* | TGGCCTTACTTGGGATTGG | CCAGTGTATATGTAGGCTCATCCA |
| *Atgl* | GGAGACCAAGTGGAACATCTCA | AATAATGTTGGCACCTGCTTCA |
| *Hsl* | TGTGGCACAGACCTCTAAAT | GGCATATCCGCTCTC |
| *Gnpat* | CGGGTTCCTGCTTTGGCCTG | CCGTTGACCACTTGTGACCT |
| *Agps* | AGGGGAGTTCAGTTCGCACC | CTCTCTAGCAGCTGCCTCAG |
| *Fads1* | CATCAGCCACTACGCGGGTC | CGGAGCCAGCTCTCCAATCA |
| *Fads2* | CAATGACTGGTTCAGCGGGC | TCAGCAACGGCTTCTCCTGG |
| *Pparα* | AGAGCCCCATCTGTCCTCTC | ACTGGTAGTCTGCAAAACCAAA |
| *Pgc1α* | GAGAATGAGGCAAACTTGCTAGCG | TGCATGGTTCTGAGTGCTAAGACC |
| *Pgc1β* | CGCTCCAGGAGACTGAATCCAG | CTTGACTACTGTCTGTGAGGC |
| *Cpt1α* | TGGCCGCATGTCAAGCCAGA | AGGAGAGCAGCACCTTCAGCGA |
| *Acox1* | CACGCACATCTTGGATGGTAGTCCG | ACGCTGGCTTCGAGTGAGGAAGTTA |
| *Vlcad* | TGCTCTGTGATAGCTGGTGC | GGCCTTGGAGATGCTTCTGA |
| *Dbp1* | ACGCCCTGGCGTTTGCAGAA | TGGCCACTGCTTTTCCGCCT |
| *β-actin* | TGTGATGGTGGGAATGGGTCAGAA | TGTGGTGCCAGATCTTCTCCATGT |
